# A Radiographic Evaluation of Uncemented Total Hip Replacements and the Role of Uncemented Implants in the Management of Hip Osteoarthritis in the Elderly Population

**DOI:** 10.7759/cureus.50487

**Published:** 2023-12-13

**Authors:** Georgios Saraglis, Joe Muscat, Yadu Shankarappa, Mohammad Sameh Mohammad Elgeweny, Mohamed Moustafa Mohamed Hussein

**Affiliations:** 1 Trauma and Orthopaedics, Bedfordshire NHS Foundation Trust, Bedford, GBR; 2 Trauma, Lister Hospital, Stevenage, GBR; 3 Orthopaedics and Trauma, Bedfordshire NHS Trust, Bedford, GBR

**Keywords:** total hip arthroplasty: tha, primary hip replacement, total hip replacement (thr), cementless total hip arthroplasty, uncemented hip replacement

## Abstract

Introduction

The idea of an uncemented, fully coated hydroxyapatite (HA) stem was introduced almost 40 years ago, aiming to achieve a solid biological fixation by preserving natural bone activity. While many studies underline the longevity of uncemented total hip replacement (THR), NHS England’s Best Practice Tariff (BPT) recommends using cemented implants in patients over the age of 69, with financial penalties when this policy is not met. At the same time, the ‘paradox’ of increased use of uncemented implants worldwide has been well described, with many surgeons using them regardless of the age group of the patient.

Materials and methods

This study focuses on the radiographic evaluation of the uncemented Pinnacle/Corail total hip replacement construct in 123 patients of all age groups who underwent an elective procedure, with a minimum radiographic follow-up of two years. Implant information (collared or non-collared stem), femur type (Dorr classification), age, gender, and revision rate were collected and radiographic analysis of the femoral stem and acetabular component was performed for the immediate post-operative, six-month, one- to two-year follow-up radiograph of all patients. We conducted a statistical analysis, dividing the patients into two groups based on age: those above or below 69 years old.

Results

There was no statistically significant difference in rates of radiographic lucency after two years with regard to the femoral component. Both collared and non-collared stems seem to perform equally well, with no significant difference detected. However, a statistically significant difference in rates of radiographic lucency of the acetabular cup was noted between the two age groups (p=0.018), with higher rates detected in the under-69-year-old age group.

Conclusion

This study demonstrates that, radiographically, the uncemented Pinnacle/Corail construct performs equally well in all age groups. In our cohort of patients, the age of the patient did not predict the osseointegration of the implant in the short-term follow-up.

## Introduction

Since their first description in 1891, total hip replacements (THR) have undergone significant evolution over the last 100 years. They are regarded as one of the most successful orthopaedic interventions [1]. McKee and Watson-Farrar first described the use of uncemented THRs in the 1950s when they implanted a cementless acetabular and femoral system into patients with reasonable results [2]. It was not, however, until the 1980s that the mechanical engineering of the materials used improved [3]. Uncemented implants rely primarily on a tight fit of the implant within the bone. Long-term survivorship depends on the biological anchoring of the implant to the bone [4]. This is achieved through osseointegration, which histologically can be defined as direct contact between the implant surface and host bone without intervening fibrous tissue [5]. Extensive research into porous coatings paved the way for the widespread use of hydroxyapatite (HA), a coating chosen for its biocompatible and osteoconductive properties. It has been used successfully for over 30 years, improving the stability and osseointegration of uncemented implants [4].

The use of uncemented implants has risen in the UK, particularly amongst younger patients [5]. The National Joint Registry’s (NJR) 19th annual report showed that 35.4% of THRs performed in 2021 were uncemented, rising steadily since 2004 (18.3%) [6]. Uncemented fixation is the most popular fixation method of choice in Canada, Australia, and the United States [7,8].

There is debate regarding the use of cemented versus uncemented THRs [3,5,7-9]. The majority of literature supports the use of cemented THRs, with evidence from various joint registry databases supporting improved patient-reported outcomes with cemented implants [3,7,10-11] and lower revision rates in older patients [10,12-14]. A concern with using cementless implants in the elderly population is osteoporosis, where failure of osseointegration and loosening of implants pose a higher risk of peri-prosthetic fracture [15].

Cementless systems offer several key benefits by negating the use of bone cement. Cement causes fragmentation and wear debris, leading to osteolysis and peri-prosthetic loosening. Cement is neither osteoconductive nor inductive and cannot remodel [12,14,15]. A rare, but potentially fatal, problem of using cement is the risk of bone cement implantation syndrome. This condition can cause severe arrhythmias and even cardiac arrest [12,16], leading to a 16-fold increase in 30-day mortality (intraoperative mortality rate up to 4.3%) [17,18-23]. In addition to removing the use of cement, cementless systems offer a shorter duration of surgery and less intraoperative blood loss [16].

Others have demonstrated good results in terms of survivorship and revision rates with the use of cementless systems within the ortho-geriatric population [5,12-14,24]. Although there is a rise in the popularity of uncemented THRs, the current guidelines by the NJR and Getting It Right First Time (GIRFT) recommend cemented or hybrid fixation in patients over 70 [6,17].

The majority of the literature focuses on patient-reported outcomes and complication rates, with relatively few papers focusing on the radiographic assessment of uncemented THRs in different age groups. The purpose of this study was to assess the radiographic stability of uncemented total hip replacements conducted in a trauma and orthopaedic unit in the UK among two specific age groups (above and below age 69). The study aimed to determine whether there was a discernible radiographic distinction in implant stability determined by measurable lucency around the implants between these age groups.

Our primary aim was to see if there was any statistically significant difference in the rates of implant radiographic lucency at two years between the two groups, those above 69 and those under 69 years of age. Our secondary aims were to compare loosening rates between collared and non-collared stems and femoral type (Dorr classification). We also collected data on any complications or revisions required.

## Materials and methods

Materials

This was a retrospective study aiming to evaluate the radiographic outcomes of the uncemented THRs. The analysis was conducted on 123 patients of all ages who underwent an elective procedure under one surgeon at one unit in the UK (Bedfordshire NHS Foundation Trust, UK) between January 2017 and April 2018 and had a minimum radiographic follow-up period of two years.

All patients had the same approach (anterolateral) and implant used Corail stem (CORAIL® Total Hip System, De Puy Synthes, Warsaw, Indiana, USA) and Pinnacle cup (PINNACLE® Hip Solutions, De Puy Synthes, Warsaw, Indiana, USA).

We collected information on the age of the patient, gender, implant used (collared or non-collared), Dorr classification for femur type [19], and revision rates. A radiographic analysis of the biologic fixation of both the femoral stem and acetabular component was performed for the immediate postoperative, six-month, one-year, and two-year follow-up radiographs of all the patients.

Methods

**Figure 1:**
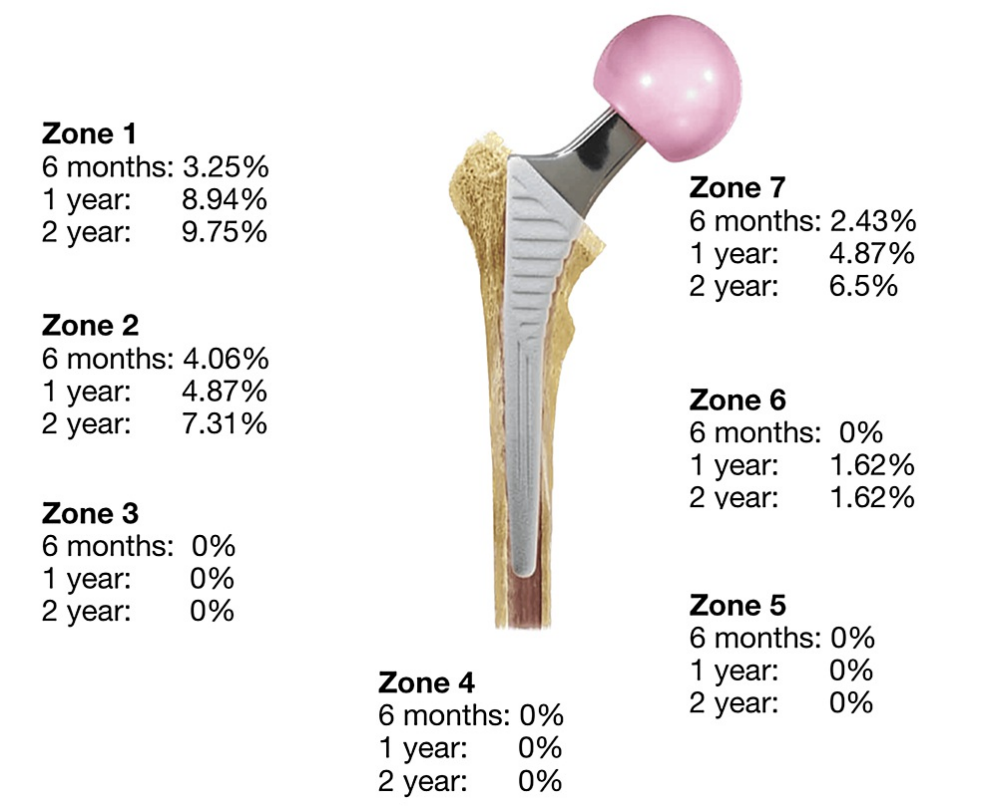
Incidence of radiographic lucency (>2 mm) around the Gruen zones of the femur.

At each of the follow-ups (post-operative, six-month, one-year and two-year), the femoral stem's Gruen zones [20] (Figure 1) and the acetabular component's DeLee and Charnley zones [21] were evaluated. We measured several radiographic parameters.

**Figure 2:**
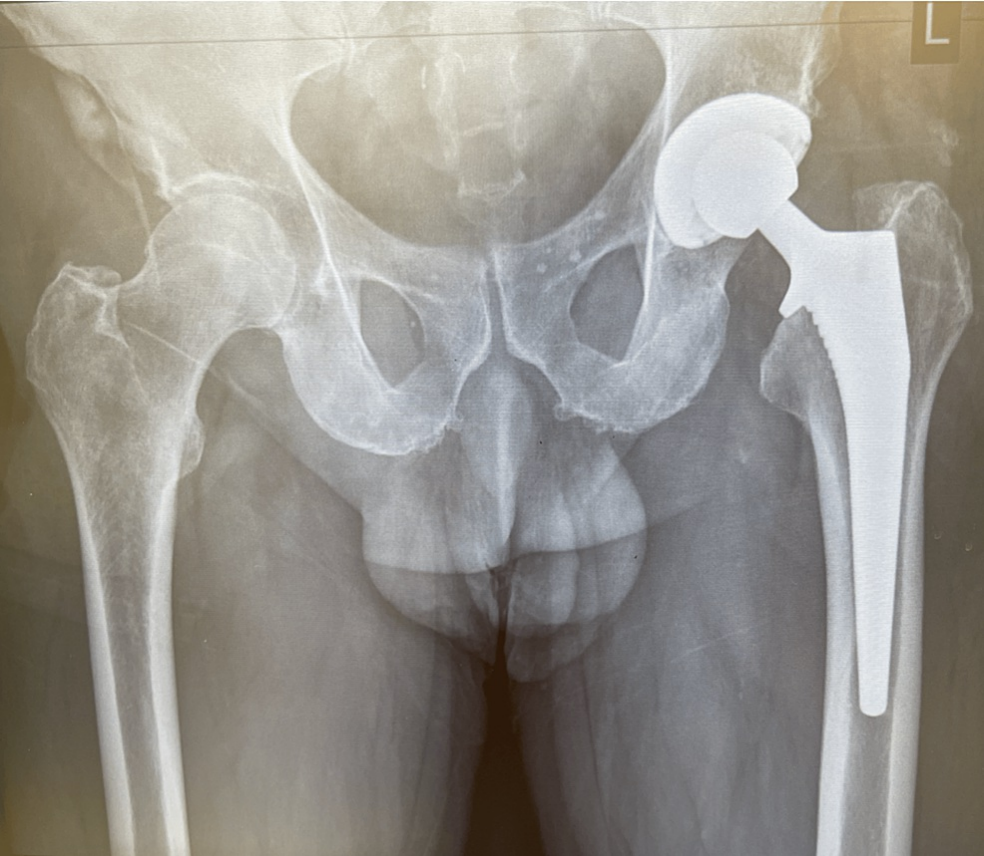
Example of uncemented collared pinnacle/corail THR, with >2 mm lucency in zone 1 of the femoral stem (12 months post-operative radiograph).

For the femoral component, signs of lucency, stem subsidence, and proximal femoral stress shielding were measured. A lucency was defined as >2 mm evident on the radiographs within a zone (Figure 2). Stem subsidence was defined as sinking of the stem greater than 5 mm.

For the acetabular component, signs of lucency and migration were measured. A lucency in this region was again defined as greater than 2 mm, and migration was defined as greater than 2 mm. Three orthopaedic surgeons, each with a minimum of five years of orthopaedic experience, performed the evaluation.

Following the collection of the data, descriptive analysis was performed, and then statistical analysis was employed (the chi-square test) to determine if there was any significant difference between the two age groups, collared versus non-collared stems, and types of femur. Descriptive analysis was performed for complications and revisions.

## Results

The overall mean age of the 123 patients was 69.3±8.6 years old. There were 58 males and 65 females. Fifty-three collared stems (43.08%) and 70 non-collared stems (56.91%) were used. Based on the Dorr classification [19], there were 31 type A, 84 type B, and 8 type C femurs.

The demographic distribution between the two groups revealed that there were 53 patients in the under-69 group with a mean age of 58.1 years and 70 patients in the over-69 group with a mean age of 76.8 years.

Primary outcome results

We noted overall femur lucency in 16 cases (13%), with 11 cases in zone 1, 9 cases in zone 2, 2 cases in zone 6, and 8 cases in zone 7. We did not observe any stem subsidence in any of the groups at two years. The results are summarised in Table 1.

**Table 1:** Radiographic evaluation of the femoral stem.

Radiographic evaluation of the femoral stem	Post-operative radiograph	6-month post-operative	1-year post-operative radiograph	2-year post-operative radiograph
Radiolucent lines (>2 mm)	0%	3.25% (4 cases)	13% (16 cases)	13% (16 cases)
Subsidence (>5 mm)	0%	0%	0%	0%
Stress shielding the proximal femur	0%	0.81% (1 case)	6.5% (8 cases)	7.31% (9 cases)

There was no statistically significant difference in the rate of femoral lucency between the two age groups (p = 0.62). In the under-69-year-old group, eight cases of femur lucency were noted (15%), and in the above-69 group, eight cases of femur lucency were also noted (11.4%) (chi-square p = 0.5). The highest incidence of radiolucent lines was noted on the one-year follow-up radiographs. Evidence of lucency was absent in immediate post-operative radiographs, with only a small incidence (3.25%) at six months. There was no further progression after two years. The results are illustrated in Figure 2. Cup lucency was noted in 12 cases (9.75%). Seven cases are in zone 1, 10 cases in zone 2, and 2 cases in zone 3. There was no evidence of cup migration. The results are summarised in Table 2.

**Table 2:** Radiographic evaluation of the acetabular component.

Radiographic evaluation of the cup (123 cases)	Post-operative radiograph (123 cases)	6-month post-operative (123 cases)	1-year post-operative (123 cases)	2-year post-operative (123 cases)
Radiolucent lines (>2 mm)	0%	2.43% (3 cases)	9.75% (12 cases)	9.75% (12 cases)
Migration (>2 mm)	0%	0%	0%	0%

There was a statistically significant difference in the rates of acetabular cup lucency between the two age groups (p = 0.018), with higher rates noted in the under-69 group. Among the 53 patients in the under-69-year-old age group, loosening of the cup radiographically was confirmed in nine cases (16.9%) preliminary in zones 1 and 2 (Charnley zones), whereas in the above-69-year-old group, only in three cases (4.28%).

Secondary outcome results

There was no statistically significant difference in lucency between collared and non-collared stems. When comparing the collared versus non-collared groups, there were five cases of femur loosening in the collared group (9.43%) and eight cases in the non-collared group (11.45%) (chi-square p = 0.72). No cases of subsidence were noted (Table 3).

**Table 3:** Secondary outcome results.

Radiographic evaluation of the femoral stem	Number of cases	Number of cases with radiographic lucency	Subsidence (>5 mm)
Collared stem	53	5 cases (9.43%)	0%
Non-collared stem	70	8 cases (11.45%)	0%
P-value	-	0.72	

Further statistical analysis was performed among the different femoral type groups (Dorr A, B, and C types) to determine if the femoral type was predisposed to radiographic lucency. Radiographic lucency was noted in six cases among the type A femurs, nine cases in the type B group, and one case among the type C femurs on the one-year post-operative radiograph, with no statistical significance among the groups (p = 0.47).

There were no noted revisions. There was one peri-prosthetic fracture, in the under-69 year age group, of the greater trochanter (Vancouver A type) around a well-fixed, non-collared, uncemented Corail stem, which was addressed using a trochanteric plate and fixed in situ.

## Discussion

This is a retrospective study comparing short-term differences in radiographic features observed with the use of the uncemented Corail/Pinnacle THA system between two age groups. Our study found there was no statistically significant difference in rates of radiographic lucency after two years with regard to the femoral component. There was, however, a statistically significant difference in rates of radiographic lucency of the acetabular cup between the age groups (p = 0.018), with higher rates actually observed in the under-69-year-old age group. There was no statistically significant difference based on the use of collared versus non-collared stems or femoral type. Additionally, there were no revision rates, and only one peri-prosthetic fracture was observed during this time. From these results, the authors conclude that there is no increased risk of loosening when using the Corail/Pinnacle system in the short term in patients over 69 years old.

After almost 40 years since the first human implantation of HA-coated implants [24], there is evidence in the recent literature supporting the use of uncemented THR implants in the elderly population [24-26]. While, worldwide, the number of uncemented THRs is rising [7], in the UK, the GIRFT recommendation for the elderly population is the use of cemented or hybrid implants, mainly over concerns of peri-prosthetic fractures, revision rates, poor bone osseointegration and the risk of loosening [27,28].

Osseointegration seems to be the key element in the survivorship of the implant and is a continuous process, occurring at any stage, in any case, regardless of the underlying bone quality and the age group of the patient [23,24]. The principle that in the elderly population with osteoporosis, the osseointegration process is restricted seems to have fallen out of favour, with studies indicating similar results between young and active and older osteoporotic patients [25].

From our study, there was no statistically significant difference in the short-term radiographic assessment of biologic fixation of the femoral stem. Interestingly, the radiographic analysis of the uncemented cup revealed a higher rate of radiolucencies in the younger age group in comparison to the elderly group, indicating that, in our cohort of patients, the age of the patient did not predict the osseointegration of the implant in the short-term follow-up.

To this note, while registered studies attribute an excellent long-term outcome to cemented implants, the number of studies supporting good mid-term results of uncemented implants in the elderly is rising [25,28,29], with our study showing similar results. The discrepancy between the registries and the evidence from the uncemented implant studies could possibly be explained by the individual type of uncemented implant used rather than the uncemented THR concept [29]. Specifically, the uncemented CORAIL stem relies on an impaction broaching system rather than an extraction system, which is frequently used by other uncemented stems. This technical difference in the broaching system, along with the biomechanics of a fully HA-coated stem, might possibly explain the excellent survivorship of the Corail/Pinnacle system with more than 20 years of long-term results [30]. When considering data published based on NJR performance, a report analysing the use of 102,823 patients receiving the collared CORAIL hip and PINNACLE cementless cup (mean age 66.5 years) had a 29% lower risk of revision when compared to all other cementless systems on the registry across all age groups [30].

Our results, in addition to those of other multiple studies [25,28,29], support the safe use of uncemented THR constructs in the elderly population. A summary of some of the key papers that support the use of uncemented THA in the elderly population is summarised in Table 3.

**Table 4:** Highlighting key papers and findings which support the use of uncemented THA in elderly population. PROMS: patient-reported outcome measures.

Paper	Method	Results/conclusion
Lewis et al. [25]	Prospectively collected data from a single-surgeon database of over 1000 uncemented THA’s (Corail/Pinnacle) performed with a mean follow-up of 5 years. Compared two groups, under 65 vs. over 65 and under 70 vs. over 70.	No significant difference in the number of revisions. PROMS were overall better than the national comparison. Those over 65/69 maintained a meaningful improvement in the Oxford Hip Score compared to younger group.
Gkagkalis et al. [28]	A prospective multicenter observational study comparing the young (under 60) and geriatric population (over 75) with mean follow up of 49.2 months with uncemented calcar guided short stem THA.	No difference in the VAS score between rest pain, load pain and satisfaction. Improved Harris Hip Score in younger patients. No difference in radiological parameters. Higher risk of periprosthetic fracture in older group. Conclusion – advanced age alone should not be a contra-indication, but those with markedly reduced bone quality and Dorr C femur are at high risk of fracture. Should therefore be reserved for Dorr A and B with good bone quality.
Zimmerer et al. [29]	Retrospectively evaluated 107 uncemented THAs in patients over 75 with mean follow up of 6.4 years.	6.3 year survivor rate of 98% with good clinical outcomes. Survivor rate is similar in literature to that of younger patients. Cementless stem showed low revision rate even in patients over 75 in mid-term. Periprosthetic fracture was not a relevant failure mechanism.

The advantages of such systems (reduced risk of bone cement implantation syndrome, shorter hospital duration, intraoperative time, and blood loss) can improve peri-operative care in such a cohort of patients. It is, however, important for the surgeon considering the use of a cementless construct to use a system that has evidence of good survivorship in the elderly group. Further long-term studies comparing the use of different uncemented broaching systems (impaction vs. extraction) may prove useful in helping determine if the difference in technique has a significant contribution to outcomes in the age group.

The authors recognise that there are limitations to this study. One main limitation is the follow-up time. Our results have only been able to generate conclusions based on a short-term follow-up (two years); further studies would be required to see if there are any medium- or long-term differences in radiographic outcomes.

Another limitation is possible inter-observer bias. Three authors collected the data, and therefore there is a possible risk of discrepancy. This was minimised by ensuring all authors were aware of the zones of measurement and the definitions of lucency/subsidence prior to data collection. The radiographic images were reviewed on the hospital Picture Archiving and Communication System (PACS) using its measuring system to allow for objective measurements to be made.

## Conclusions

Our study found there was no statistically significant difference in rates of loosening of the femoral component based on age or the use of collared versus non-collared stems. Despite the statistically significant higher incidence of radiographic lucency of the acetabular cup in the under-69-year-old age group (p = 0.018), no clinical correlation was noted. Additionally, there were no revision rates, and only one peri-prosthetic fracture was observed during this time. Our results suggest that there is no correlation between age and radiographic loosening rates in the short-term follow-up.

The uncemented Pinnacle/Corail construct seems to perform equally well in all age groups at short-term follow-up. The age of the patient does not seem to predict the osseointegration of the implant during the short-term follow-up.
